# Epidemiological study of *Toxocara canis* infection in dogs in selected regions of Egypt

**DOI:** 10.3389/fvets.2026.1895478

**Published:** 2026-07-31

**Authors:** Hattan S. Gattan, Mohamed Marzok, Abdelfattah Selim, Mohammed H. Alruhaili, Hesham Ismail, Adel I. Almubarak

**Affiliations:** 1 Department of Medical Laboratory Sciences, Faculty of Applied Medical Sciences, King Abdulaziz University, Jeddah, Saudi Arabia; 2 Special Infectious Agents Unit, King Fahad Medical Research Center, King Abdulaziz University, Jeddah, Saudi Arabia; 3 Department of Clinical Sciences, College of Veterinary Medicine, King Faisal University, Al-Ahsa, Saudi Arabia; 4 Department of Animal Medicine (Infectious Diseases), Faculty of Veterinary Medicine, Benha University, Toukh, Egypt; 5 Department of Clinical Microbiology and Immunology, Faculty of Medicine, King Abdulaziz University, Jeddah, Saudi Arabia; 6 Department of Public Health, College of Veterinary Medicine, King Faisal University, Al-Ahsa, Saudi Arabia

**Keywords:** deworming, dogs, Egypt, risk factors, seroprevalence, *Toxocara canis*, zoonosis

## Abstract

**Background:**

Toxocariasis caused by *Toxocara canis* is a common parasitic disease in dogs with important zoonotic implications. Although fecal examination is traditionally used for diagnosis, it has limitations in detecting prepatent, low-intensity, or intermittent infections. Therefore, serological testing was selected in this study as a more sensitive and time-efficient approach for assessing exposure at the population level. This study aimed to determine the seroprevalence of *T. canis* infection and identify associated risk factors among dogs in four Egyptian governorates.

**Methods:**

A total of 409 dogs from Cairo, Qalyubia, Kafr El-Sheikh, and Gharbia were sampled. Serum samples were tested for anti-*T. canis* IgG antibodies using a sensitive immunoassay. Epidemiological and management-related risk factors, including age, sex, feeding practices, housing conditions, cohabitation with cats, coprophagy, and deworming history, were recorded and analyzed using univariate and multivariate logistic regression models.

**Results:**

The overall seroprevalence was 25.9% (106/409; 95% CI: 21.91–30.38%), with the highest prevalence in Cairo (32.2%) and the lowest in Qalyubia (19%). Dogs older than 4 years showed the highest seropositivity (37.4%). Seroprevalence was significantly higher among dogs fed homemade food (36%), living outdoors (41.5%), cohabiting with cats (32.4%), exhibiting coprophagy (30.4%), and those without regular deworming (33%). No statistically significant differences were observed between male and female dogs. Multivariate logistic regression identified older age, outdoor living, cohabitation with cats, coprophagy, homemade diet, and irregular deworming as significant risk factors for infection.

**Conclusion:**

The findings demonstrate substantial exposure of dogs to *T. canis* in the studied regions and highlight the cumulative impact of environmental and management-related risk factors. The use of serology provided a broader estimate of exposure than fecal examination alone. Regular deworming, improved feeding practices, and environmental hygiene are essential to reduce infection risk and minimize zoonotic transmission.

## 1 Introduction

Toxocariasis is a zoonotic parasitic disease caused by the ascarid roundworms *Toxocara canis* and *Toxocara cati*, which are recognized as the most prevalent intestinal helminths infecting domestic dogs and cats worldwide (1, 2). These parasites are of major veterinary and public health importance, as they are responsible for the development of Visceral Larva Migrans (VLM) and Ocular Larva Migrans (OLM) in humans (3). In Egypt, canine toxocariasis remains an important yet underrecognized zoonotic infection due to the large population of owned, shelter, and stray dogs, coupled with environmental contamination by infective eggs in public spaces. Several Egyptian studies have reported the presence of *Toxocara* eggs in soil and documented exposure in both animals and humans, indicating ongoing transmission and a persistent public health risk, particularly among children and immunocompromised individuals. Despite their widespread distribution and significant health impact, toxocariasis and its clinical manifestations are frequently classified as neglected diseases, receiving limited attention in surveillance, diagnosis, and control strategies (4–8).

In dogs, the life cycle of *T. canis* is age dependent. In young puppies, the parasite completes its full developmental cycle, which includes a pulmonary migratory phase before maturation into adult worms residing in the lumen of the small intestine (9, 10). These adult worms produce large numbers of eggs that are shed in the feces, contributing substantially to environmental contamination. In contrast, in adult dogs, ingested larvae typically do not develop into adult worms. Instead, they migrate and become arrested in various tissues, such as the liver, lungs, and musculature, where they persist in a somatic state. As a consequence, coprological examination of fecal samples from mature dogs often yields negative results, making egg detection unreliable and leading to an underestimation of infection prevalence in this age group (7, 11, 12).

In pregnant bitches, somatically arrested larvae of *T. canis* become reactivated and migrate transplacentally to the developing fetuses, resulting in puppies being born already infected (13). These congenitally infected pups subsequently harbor intestinal adult worms and are capable of shedding large numbers of parasite eggs during the first months of life, thereby playing a critical role in environmental contamination and transmission dynamics (14, 15).

The persistently high prevalence of *T. canis* infection among stray dogs, owned pets, and human populations in many regions worldwide can be attributed to a combination of socio-environmental and public health factors (16, 17). *T. canis* infection remains highly prevalent among stray and owned dogs, representing an important One Health challenge due to its close association with environmental contamination and human exposure (18). Dogs serve as the primary definitive hosts, shedding large numbers of eggs in feces that contaminate soil, water, and public spaces, thereby increasing the risk of accidental human infection, particularly among children and immunocompromised individuals (19). Human exposure to infective eggs may lead to serious zoonotic manifestations, including visceral larva migrans and ocular larva migrans. The continued transmission of *T. canis* in both canine and human populations is influenced by multiple interconnected socio-environmental factors, including the high density of stray and free-roaming dogs, inadequate responsible pet ownership, poor fecal waste management in public areas, and limited public awareness regarding zoonotic parasitic diseases. Additionally, insufficient coordination between veterinary and public health sectors further limits effective prevention and control measures, emphasizing the importance of integrated One Health strategies for toxocariasis control (20).

Numerous studies have investigated the prevalence of *T. canis* egg shedding in canine fecal samples across different geographic regions, reporting highly variable results. For instance, Haridy et al. (21) documented a positivity rate of 9.83% among 3,000 owned dogs examined in Cairo, Egypt. In the United States, reported prevalence rates range widely from as low as 2% to as high as 79%, reflecting substantial differences in study populations, diagnostic methods, and environmental conditions (22). Similarly, studies conducted in Brazil have demonstrated considerable variability, with positivity rates of 9.3, 18.9, and 44.3% reported by Scaini et al. (23), Labruna et al. (24) and Chieffi and Müller (25), respectively.

It is important to note that several of these investigations may underestimate the true prevalence of *T. canis* infection in canine populations. Conventional coprological examination has limited sensitivity, as it fails to detect infections in a substantial proportion of infected dogs, particularly adult animals (26). Infected adult male dogs generally do not shed eggs, whereas female dogs represent a critical epidemiological reservoir. Female canids are capable of transmitting the parasite congenitally to their offspring and may resume egg shedding following parturition, thereby sustaining environmental contamination and transmission cycles (27). However, serological assays also have inherent limitations. The detection of anti-*T. canis* antibodies reflects exposure to the parasite but cannot reliably distinguish between active current infection and past infection (28). Antibodies may persist for prolonged periods even after parasite clearance, which may lead to overestimation of active infection rates. Additionally, serological cross-reactivity with other helminths may occasionally affect specificity. Therefore, serological findings should be interpreted cautiously and, whenever possible, complemented with clinical, epidemiological, and parasitological data to provide a more comprehensive assessment of infection status (29–31).

Young companion animals are widely recognized as a significant public health concern, as they play a central role in the transmission of visceral and ocular larva migrans to humans, including in developed regions. Human infection occurs accidentally through the ingestion of embryonated eggs containing infective larvae. Once ingested, the larvae hatch and migrate through various organs and tissues, including the eyes, without completing their development into adult worms. The resulting clinical manifestations are highly variable and depend on several factors, such as the infective dose, the tissue tropism of the larvae, and the host’s immune response (32, 33).

Although toxocariasis is rarely fatal, it is associated with substantial morbidity. Common clinical outcomes include asthma-like respiratory symptoms and visual impairment resulting from ocular involvement. In some cases, larval migration to the central nervous system has been reported, leading to neurological manifestations such as seizures, neuropsychiatric disturbances, or encephalopathy (34). These clinical conditions impose a measurable burden on healthcare systems due to the costs associated with diagnosis, clinical management, and long-term treatment (35).

As with many parasitic infections, host-related factors such as age, immune status, genetic predisposition, and prior exposure may substantially influence susceptibility to Toxocariasis and the resulting clinical outcome. In addition to symptomatic disease, asymptomatic *Toxocara* infections may induce significant immunomodulatory effects in the host. These infections have been associated with elevated total IgE levels, peripheral eosinophilia, and altered immune regulation, which may increase susceptibility to allergic conditions such as asthma and other atopic disorders. Such host-dependent immune responses may affect parasite persistence, disease severity, and transmission dynamics. These immunological alterations further emphasize the veterinary and public health significance of toxocariasis, even in individuals or animals lacking overt clinical signs (36, 37).

The present study was conducted in four Egyptian governorates (Cairo, Qalyubia, Kafr El-Sheikh, and Gharbia) selected to represent diverse ecological, demographic, and dog-management conditions across Egypt. Cairo was included because of its high population density, extensive urbanization, and large number of owned and stray dogs, all of which may increase environmental contamination and zoonotic risk. Qalyubia, Gharbia, and Kafr El-Sheikh were selected to represent mixed urban–rural and agricultural settings where close contact among dogs, livestock, and humans may facilitate parasite transmission. This geographic diversity allowed a broader assessment of *T. canis* epidemiology and associated risk factors under varying environmental and management conditions.

## 2 Materials and methods

### 2.1 Ethical statement

All experimental procedures involving animals were conducted in accordance with the ethical standards and guidelines approved by Ethical Committee of the Faculty of Medicine, Benha University. Verbal informed consent was obtained from all dog owners before administering the questionnaire and collecting samples from their animals. All procedures were carried out in accordance with applicable national and international ethical guidelines for animal research.

### 2.2 Study area

**Figure 1:**
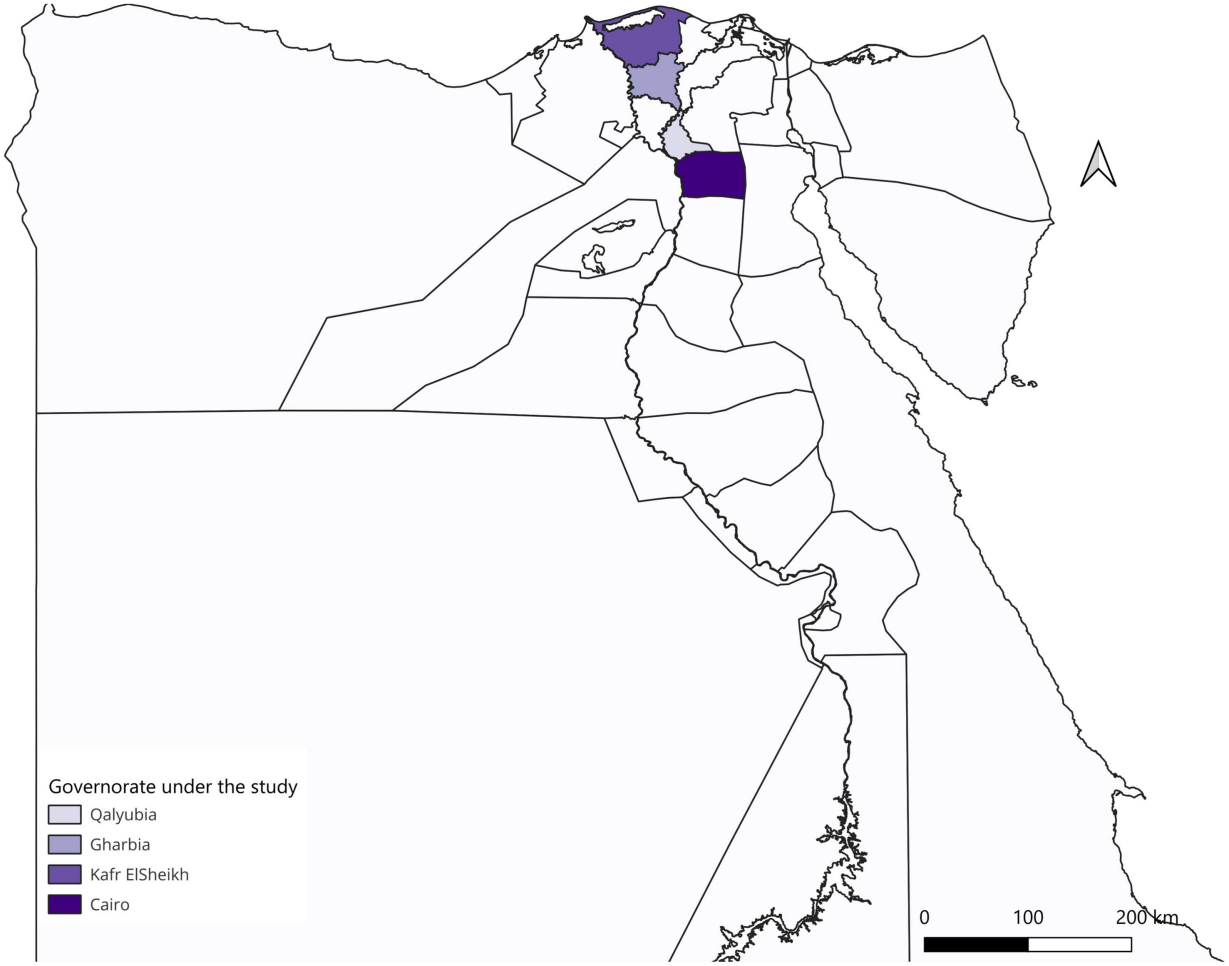
MAP showing governorates under the study. Created using QGIS.

A cross-sectional study was conducted from January to December 2025 to determine the prevalence of *Toxocara canis* infection in dogs across four Egyptian governorates: Cairo, Qalyubia, Kafr El-Sheikh, and Gharbia (Figure 1). These governorates are located within the Nile Delta region of northern Egypt, a low-lying and densely populated area characterized by fertile agricultural lands and an extensive network of irrigation canals. Geographically, the study area lies approximately between 30°–31° N latitude and 30°–31° E longitude. Its proximity to the Mediterranean Sea plays a significant role in shaping the local climate, particularly influencing temperature patterns and relative humidity.

Cairo and Qalyubia governorates experience a hot desert climate (BWh, Köppen classification) characterized by long, hot summers and mild winters. During summer months (May–September), temperatures commonly range between 30 and 40 °C and may exceed 42 °C during heat waves, while winter temperatures (December–February) are generally mild, averaging between 10 and 20 °C. Rainfall is low and irregular, occurring mainly in winter, with an annual average of approximately 20–50 mm. Relative humidity is moderate to high, particularly in Qalyubia due to extensive agricultural activities and irrigation canals, whereas Cairo is influenced by urban heat and lower humidity levels. Both governorates are affected by seasonal Khamsin winds in spring, which bring hot, dry conditions and occasional dust storms. The proximity of the Nile Delta and the Mediterranean Sea contributes to moderating winter temperatures and increasing humidity in the region.

Kafr El-Sheikh and Gharbia governorates are located in the central and northern parts of the Nile Delta and experience a Mediterranean-influenced desert climate characterized by hot summers and mild, relatively wetter winters. During summer (May–September), temperatures generally range between 28 and 38 °C, while winter temperatures (December–February) are moderate, averaging between 10 and 18 °C. Rainfall is low but higher than in southern Delta regions, occurring mainly in winter and ranging from approximately 50 to 100 mm annually, particularly in Kafr El-Sheikh due to its proximity to the Mediterranean Sea. Relative humidity is relatively high throughout the year, supported by extensive agricultural lands and irrigation networks. Both governorates are occasionally affected by seasonal Khamsin winds during spring, bringing warm, dry air and dust storms. The climatic conditions favor agricultural productivity but also support the survival and transmission of many parasitic and infectious agents.

### 2.3 Sample size and sampling

The sample size for this study was calculated using a prevalence-based formula that takes into account the expected prevalence of *T. canis* infection in dogs, the desired confidence level, and the acceptable margin of error. In the absence of reliable previous data, an expected prevalence of 50% was assumed to ensure the maximum sample size. Using a 95% confidence interval (*Z* = 1.96) and a precision of 5% (*d* = 0.05), the minimum required sample size was estimated according to the following formula:

$$n=\frac{Z^{2}\times P(1-P)}{d^{2}}$$

Where *n* is the required sample size, *Z* is the Z-score for a 95% confidence level (1.96), *P* is the expected prevalence (0.50), and *d* is the desired precision (0.05). Based on this calculation, a total of 409 dogs were included in the study across the four governorates (Cairo, Qalyubia, Kafr El-Sheikh, and Gharbia) to ensure adequate statistical power.

Approximately 5 mL of blood was collected from the forearm vein by venipuncture into plain tubes without anticoagulant and allowed to clot at room temperature for 30 min. Samples were centrifuged at 1500 rpm for 10 min to separate the serum, which was then collected and stored at −20 °C until serological testing.

### 2.4 Questionnaire

During sampling, a structured questionnaire was completed by dog owners to collect information on potential risk factors associated with *Toxocara canis* infection. The questionnaire recorded data on the dog’s locality (Cairo, Qalyubia, Kafr El-Sheikh, or Gharbia), sex (male or female), and age (6–12 months, 1–2 years, 2–4 years, and >4 years). Additional variables included homemade food consumption (yes/no), lifestyle (indoor or outdoor), cohabitation with cats (yes/no), coprophagy (yes/no), and deworming history (yes/no). These variables were subsequently analyzed for their association with the prevalence of *T. canis* infection using descriptive statistics and multivariable logistic regression models.

### 2.5 Serological testing

Anti-*Toxocara* IgG antibodies were detected using a commercial Enzyme-Linked Immunosorbent Assay (ELISA) kit (NovaTec Immunodiagnostica GmbH, Dietzenbach, Germany), which exhibits high diagnostic sensitivity and specificity (>95%) and is considered a reference method for the serodiagnosis of toxocariasis.

Serum samples were diluted 1:100 with IgG sample diluent, and 100 μL of each diluted sample was dispensed duplicate into microplate wells and incubated at 37 °C for 1 h. After three washing cycles, 100 μL of *T. canis* protein A horseradish peroxidase (HRP) conjugate was added to each well and incubated at 37 °C for 30 min. Color development was achieved by adding 100 μL of tetramethylbenzidine (TMB) substrate and incubating for 15 min at room temperature. The reaction was terminated by adding 100 μL of stop solution, and optical density (OD) was measured at 450/620 nm using a microplate reader.

As recommended by the manufacturer, the absorbance values for the cut-off control ranged from 0.150 to 1.30, negative controls were below 0.200 the cut-off, and positive controls were above the cut-off.

### 2.6 Statistical analysis

All statistical analyses were performed using SPSS version 24 (IBM Corp., Armonk, NY, USA). Initially, descriptive statistics were calculated to summarize the prevalence of *T. canis* infection among dogs across the different categorical variables, including locality, sex, age, homemade food ingestion, lifestyle, cohabitation with cats, coprophagy, and deworming status.

Differences in prevalence between these categorical variables were assessed using the Chi-square (*χ*^2^) test. Variables with *p*-values ≤ 0.2 in the univariate analysis were subsequently included in multivariate logistic regression models to control for potential confounding effects and to quantify the strength of association between each risk factor and infection status. The results of the logistic regression were expressed as odds ratios (ORs) with 95% confidence intervals (95% CI), providing a measure of the likelihood of infection associated with each risk factor relative to the reference category. Statistical significance was defined as a *p*-value ≤ 0.05. Model fit was evaluated using standard goodness-of-fit tests, and multicollinearity between independent variables was assessed prior to inclusion in the regression models to ensure reliable estimates.

## 3 Results

A total of 409 dogs were examined across the four governorates, and the overall seroprevalence of *T. canis* was 25.9% (106/409). The highest prevalence was observed in Cairo (32.2%), while the lowest was recorded in Qalyubia (19%) (Table 1). Sex had no significant effect (*p* = 0.202) on seroprevalence, although males showed a slightly higher rate (28.3%) compared to females (22.2%). In contrast, several factors were significantly associated with *T. canis* infection, including age (*p* = 0.002), homemade food ingestion (*p* < 0.0001), lifestyle (*p* < 0.0001), cohabitation with cats (*p* < 0.0001), coprophagy (*p* = 0.003), and deworming status (*p* = 0.001). Seroprevalence was highest in dogs older than 4 years (37.4%), those fed homemade food (36%), outdoor-housed dogs (41.5%), dogs cohabiting with cats (32.4%), coprophagic dogs (30.4%), and dogs that had not received deworming treatment (33%). These findings indicate that both environmental and management factors contribute to the distribution of *T. canis* infection in the studied population, Table 1.

**Table 1:** Seroprevalence of *Toxocara canis* in relation to different factors in dogs.

Location	No of examined dogs	No of positive	%	95%CI	Statistic
Cairo	115	37	32.2	24.33–41.16	
Qalyubia	105	20	19.0	12.68–27.6	*χ*^2^ = 6.378 *df* = 3 *p* = 0.095
Kafr ElSheikh	94	28	29.8	21.49–39.68	
Gharbia	95	21	22.1	14.94–31.45	
Age					
6–12 months	30	2	6.7	1.85–21.33	*χ*^2^ = 14.453 *df* = 3 *p* = 0.002*
1–2 years	65	12	18.5	10.89–29.55	
2–<4 years	215	55	25.6	20.21–31.81	
>4 years	99	37	37.4	28.48–47.2	
Sex					
Male	251	71	28.3	23.08–34.16	*χ*^2^ = 1.901 *df* = 1 *p* = 0.202
Female	158	35	22.2	16.38–29.24	
Homemade food ingestion					
Yes	164	59	36.0	29.03–43.57	*χ*^2^ = 14.427 *df* = 1 *p* < 0.0001*
No	245	47	19.2	14.74–24.57	
Life style					
Indoor	233	33	14.2	10.26–19.22	*χ*^2^ = 38.960 *df* = 1 *p* < 0.0001*
Outdoor	176	73	41.5	34.46–48.86	
Cohabitation with cats					
Yes	238	77	32.4	26.73–38.53	*χ*^2^ = 12.281 *df* = 1 *p* < 0.0001*
No	171	29	17.0	12.08–23.3	
Coprophagy					
Yes	273	83	30.4	25.25–36.1	*χ*^2^ = 8.605 *df* = 1 *p* = 0.003*
No	136	23	16.9	9.59–20.28	
Deworming					
Yes	194	35	18.0	13.27–24.05	*χ*^2^ = 11.922 *df* = 1 *p* = 0.001*
No	215	71	33.0	27.08–39.56	
Total	409	106	25.9	21.91–30.38	

^*^Results are significant if *p* < 0.05.

Multivariate logistic regression analysis indicated that several factors significantly increased the risk of *T. canis* infection in dogs (Table 2). Dogs older than 4 years were 5.4 times more likely to be infected than younger dogs (OR = 5.4, 95%CI: 1.13–26.22). Dogs fed homemade food had a 2.1-fold higher risk (OR = 2.1, 95%CI: 1.26–3.54), while outdoor-housed dogs showed a 5.4-fold increase in infection likelihood (OR = 5.4, 95%CI: 3.22–9.23). Additionally, dogs living with cats had a 2.8-fold higher risk (OR = 2.8, 95%CI: 1.61–4.98), and coprophagic dogs and those not receiving antiparasitic treatment exhibited 2.5-fold (OR = 2.5, 95%CI: 1.40–4.58) and 1.6-fold higher odds of infection (OR = 1.6, 95%CI: 0.95–2.68), respectively.

**Table 2:** Multivariate logistic regression analysis for risk factors associated with *Toxocara canis* infection in dogs.

Variable	B^a^	SE^b^	OR^c^	95% CI for OR		*p* value
				Lower	Upper	
Age						
1–2 years	0.760	0.844	2.1	0.41	11.19	0.368
2–<4 years	0.970	0.788	2.6	0.56	12.35	0.218
>4 years	1.695	0.802	5.4	1.13	26.22	0.034
Homemade food ingestion						
Yes	0.748	0.264	2.1	1.26	3.54	0.005
Life style						
Outdoor	1.695	0.269	5.4	3.22	9.23	<0.0001
Cohabitation with cats						
Yes	1.041	0.288	2.8	1.61	4.98	<0.0001
Coprophagy						
Yes	0.929	0.303	2.5	1.40	4.58	0.002
Deworming						
No	0.467	0.266	1.6	0.95	2.68	0.029

B, Logistic regression coefficient; SE, standard error; OR, odds ratio; 95%CI, confidence interval.

## 4 Discussion

In most investigations of canine toxocariasis, infection is commonly diagnosed through the detection of parasite eggs in fecal samples (25, 38). Nevertheless, several studies have employed serological detection of anti-*Toxocara canis* IgG antibodies as an indicator of infection, reporting prevalence rates of 53% in Latin America (39), 33.6% in Italy (20), 66.7% in Mexico City (40), and 0.9% in Chuncheon, South Korea (41).

In the present study, seroprevalence was used as an indicator of infection to evaluate the risk factors associated with toxocariasis in the canine population. The immunoassay employed is more sensitive and less time-consuming than conventional parasitological examination of fecal samples for the diagnosis of *T. canis* infection. However, antibody detection indicates exposure to *T. canis* but cannot differentiate active infection from past infection, as antibodies may persist after parasite clearance.

The relatively high seroprevalence of *Toxocara* infection observed in the present study (25.9, 95% CI: 21.91–30.38%) indicates that toxocariasis is prevalent among pet-owning populations. These findings are consistent to those reported in Mexico (26.2%) (42). However, the observed seroprevalence was higher than that reported in Slovakia (3.7%), the Netherlands (10.7%), Thailand (6.0%), the Russian Federation (1.1%), and Austria (12.9%) (42–46). In contrast, the prevalence recorded in this study was lower than that reported in Taiwan (46.0%), the Philippines (49.0%), and Ghana (53.5%) (47–49).

These discrepancies may also be attributed to variations in the study populations and age groups of participants, which can influence the observed seroprevalence of toxocariasis. Additionally, differences in laboratory techniques and the use of various commercial diagnostic kits play a significant role in the variability of *Toxocara* infection prevalence reported across different studies (50–53). In general, the prevalence of *Toxocara* infection is influenced by multiple factors, including geographic location, climatic conditions, cultural practices, and various socioeconomic determinants.

No statistically significant differences in *Toxocara* seroprevalence were observed among the studied governorates, indicating a relatively similar level of exposure across all regions. This homogeneous distribution may reflect the widespread environmental contamination with infective *T. canis* eggs, their prolonged survival under favorable environmental conditions, and common transmission risk factors such as high stray dog populations, inadequate deworming practices, and frequent exposure to contaminated soil or fecal matter, all of which contribute to sustaining the parasite’s life cycle and transmission (54–59).

An age-wise analysis demonstrated that the seroprevalence of *Toxocara* infection increased with advancing age, with dogs older than 4 years exhibiting the highest rate of 37.4%. This pattern indicates cumulative exposure to infective eggs over time, reflecting the progressive nature of environmental contact and risk. These findings are in agreement with previous studies, which also reported higher seropositivity rates among older dogs (60–62). Older animals are more likely to harbor chronic infections, as *T. canis* larvae can persist in host tissues for extended periods, thereby sustaining detectable antibody levels and contributing to continued seropositivity within the population (11, 63).

Sex did not have a significant effect on seroprevalence, which is consistent with previous studies reporting no substantial differences between male and female dogs (61, 64). Nevertheless, some investigations have documented marginally higher infection rates in males, potentially due to increased roaming behavior and greater exposure to contaminated environments (62, 65, 66).

Interestingly, the seroprevalence of *T. canis* was significantly higher in dogs fed homemade food, a finding that aligns with the results reported by Zibaei et al. (67). This association may be explained by increased exposure to infective stages of the parasite through improperly handled food ingredients. Homemade diets may include raw or undercooked meat, offal, or contaminated vegetables, which can serve as potential sources of parasite eggs or larvae. In addition, food preparation and storage under suboptimal hygienic conditions may increase the likelihood of contamination. These factors may collectively contribute to a higher risk of exposure in dogs consuming homemade diets (68, 69).

The seroprevalence of *T. canis* was higher among dogs living outdoors, consistent with the findings of Zibaei et al. (67). This consistent association highlights the outdoor environment as a significant risk factor for infection, as outdoor dogs are exposed for extended periods to potential sources of *T. canis*, including contaminated soil, cat feces, and possibly infected small mammals or birds, regardless of the dogs’ primary function or role (70–74).

It is notable that seroprevalence was significantly higher in dogs living in close contact with cats. This finding is consistent with the results of the ordered logistic regression analysis, which showed elevated antibody titers in dogs cohabiting with felines. The agreement between these analyses highlights that feline cohabitation is an important risk factor for increased *T. canis* infection (75). Following excretion in the feces of infected cats, *T. canis* oocysts can contaminate the surrounding environment. These oocysts tend to accumulate near or within areas where cats frequently defecate, thereby creating localized hotspots of contamination. Consequently, dogs sharing living spaces with cats are more likely to be exposed to these contaminated environments, which increases their risk of infection (76).

Coprophagy showed a similar association with *T. canis* seropositivity, as dogs exhibiting this behavior had higher antibody titres. Coprophagy, which can involve ingestion of feces from dogs, cats, or other species, increases the risk of infection, particularly when dogs consume feces from cats that may contain large numbers of oocysts (26, 77).

The deworming status of dogs was found to be a significant determinant of *Toxocara* infection risk. Dogs that had not received regular anthelmintic treatment exhibited substantially higher infection rates compared to those that were dewormed, which consistent with findings of Said et al. (62). This finding underscores the crucial role of preventive veterinary interventions, as routine deworming effectively reduces the parasite burden, limits environmental contamination with infective eggs, and consequently decreases the likelihood of transmission within canine populations (61).

## 5 Conclusion

This study demonstrated a considerable seroprevalence of *T. canis* (25.9%) among dogs in the investigated Egyptian governorates, confirming its ongoing epidemiological and zoonotic importance. Older age, outdoor living, cohabitation with cats, coprophagy, homemade feeding, and irregular deworming were identified as significant risk factors for infection. However, the use of serological testing represents a limitation, as it cannot distinguish active from past infections. Future studies integrating serological and parasitological approaches, along with broader surveillance, are recommended to improve understanding and strengthen control strategies for canine toxocariasis.

## Data Availability

The raw data supporting the conclusions of this article will be made available by the authors, without undue reservation.
